# Our Journal

**Published:** 1848

**Authors:** 


### ARTICLE IV.

### OUR JOURNAL.

This number is the first of the fifth volume since the work commenced, and begins a new series under a new title. In looking back over the labors of the past, it is highly gratifying to see with what liberality the work has been sustained by the patronage and contributions of the profession of the North-West. And in this last respect particularly have we reason to be thankful, for it has enabled us to furnish to our readers a larger amount of original matter than is given in most of our cotemporaries, of the same size. These favors, we have assurances, will be continued, and, we hope, still more abundantly.

The numerous Medical Societies of the North-West ought to furnish from their archives many highly valuable and interesting papers and reports for publication. This would add interest to the exercises of the associations, by being an incentive to greater research and closer observation in the production of articles, when understood that, if deemed worthy by the society, they are to go before the public, and be permanently placed upon record.

Those of our subscribers who are members of medical societies, will please, for us, tender such associations the use of our Journal for the publication of any articles read before them that may be deemed of interest to the profession.

As there has been, of late, some just ground of complaint against us for a want of punctuality in the time of our issues, which has arisen from our not having a publisher who was interrested in the work, it affords us great pleasure to be able to assure our patrons that they may expect greater regularity hereafter—Mr. John W. Duzan, a practical printer, who has few superiors as a workman in the west, having become interested in the Journal as publisher. E.

